# Erroneous attribution of relevant transcription factor binding sites despite successful prediction of *cis*-regulatory modules

**DOI:** 10.1186/1471-2164-12-578

**Published:** 2011-11-25

**Authors:** Marc S Halfon, Qianqian Zhu, Elizabeth R Brennan, Yiyun Zhou

**Affiliations:** 1 Department of Biochemistry, State University of New York at Buffalo, Buffalo, NY 14214 USA; 2 Department of Biological Sciences, State University of New York at Buffalo, Buffalo, NY 14260 USA; 3 New York State Center of Excellence in Bioinformatics and the Life Sciences, Buffalo NY 14203 USA; 4 Department of Molecular and Cellular Biology, Roswell Park Cancer Institute, Buffalo NY 14263 USA; 5 Center for Human Genome Variation, Duke University, Durham, NC 27708, USA

## Abstract

**Background:**

*Cis*-regulatory modules are bound by transcription factors to regulate gene expression. Characterizing these DNA sequences is central to understanding gene regulatory networks and gaining insight into mechanisms of transcriptional regulation, but genome-scale regulatory module discovery remains a challenge. One popular approach is to scan the genome for clusters of transcription factor binding sites, especially those conserved in related species. When such approaches are successful, it is typically assumed that the activity of the modules is mediated by the identified binding sites and their cognate transcription factors. However, the validity of this assumption is often not assessed.

**Results:**

We successfully predicted five new *cis*-regulatory modules by combining binding site identification with sequence conservation and compared these to unsuccessful predictions from a related approach not utilizing sequence conservation. Despite greatly improved predictive success, the positive set had similar degrees of sequence and binding site conservation as the negative set. We explored the reasons for this by mutagenizing putative binding sites in three *cis*-regulatory modules. A large proportion of the tested sites had little or no demonstrable role in mediating regulatory element activity. Examination of loss-of-function mutants also showed that some transcription factors supposedly binding to the modules are not required for their function.

**Conclusions:**

Our results raise important questions about interpreting regulatory module predictions obtained by finding clusters of conserved binding sites. Attribution of function to these sites and their cognate transcription factors may be incorrect even when modules are successfully identified. Our study underscores the importance of empirical validation of computational results even when these results are in line with expectation.

## Background

Developmental control of gene expression depends on the activity of transcriptional enhancers, or "*cis*-regulatory modules" (CRMs) [reviewed by [1,2]]. These regulatory sequences coordinate the binding of sequence-specific transcription factors and can stimulate transcription of their target genes irrespective of distance, location (5' or 3'), and orientation relative to the transcription start site. A CRM typically contains one or more binding sites for several different transcription factors (TFs), both activators and repressors. Efficient discovery of CRMs in the genome has proven challenging, although significant progress has been made recently with respect to both experimental and bioinformatics approaches [3]. As with many genomic techniques, however, in silico results have accumulated much faster than those from in vivo validation, and many assumptions about both empirically and computationally identified enhancers--including about their functions and the functions of their component binding sites--remain to be rigorously assessed.

Methods for computational enhancer discovery have commonly first attempted to identify the constituent transcription factor binding sites (TFBSs) based on the logic "find the binding sites, find the enhancer" [see reviews by [3-5]]. A serious complicating factor, however, is the fact that TFBS identification carries an unsuitably high false-positive rate in terms of predicting functional binding sites. Noting that this rate can be as high as 1000-fold, Wasserman and Sandelin [6] have coined the "futility theorem--that essentially all predicted TFBSs will have no functional role." As a result, attempting to identify CRMs solely based on prediction of TFBSs for a single factor tends to be unsuccessful, save for relatively rare exceptions in which the TFBSs are numerous and tightly clustered [e.g. [7,8]]. Two basic strategies have been used to overcome this problem. One is to assess the conservation of TFBSs across species, on the theory that important sites will be well conserved. The second is to search simultaneously for a cluster of several different TFBSs that comprise the "transcriptional code" for a given pattern of gene expression. This is based on the simple idea that the probability of finding a specific *combination *of TFBSs within a small sequence window is much smaller (and therefore more significant) than that of finding a single site. While details and implementations differ among methods, on the whole, approaches based on either of these strategies have been able to achieve moderate-to-good success, with the best results tending to occur when the two approaches are combined [e.g. [9-14]]. Nevertheless, where sufficient in vivo validation has been performed, false-positive prediction rates are seen to remain substantial even for some of the best-performing motif-based methods. Despite this, the reasons underlying both successful and false predictions have not been extensively explored.

That false-positive rates remain a problem is perhaps not surprising, given the many factors that could contribute to both real and apparent incorrect predictions. For example, poor prediction of the component TFBSs would lead to a false-positive result, while limitations of the reporter gene assay--incompatible promoters or failure to detect weak activity, for instance--would cause a true prediction to appear as a false-positive upon in vivo validation. True-positive results, on the other hand, are almost invariably assumed to be true as a result of having the correct underlying model, i.e., that the TFBS motifs used as input to the computational search reflect relevant binding sites essential for the activity of the discovered CRM. It is also generally accepted that these TFBSs are bound by the presumed cognate TF. However, in most cases these assumptions are not in fact tested (for example, by mutagenesis of individual TFBSs). We previously showed that another common assumption--that if a predicted CRM maps adjacent to a gene with the expected expression pattern, it must be a valid prediction--does not hold up well to empirical testing [11]. Here, we test the assumption that successful CRM prediction implies prior accurate identification of component TFs and TFBSs by undertaking extensive TFBS mutagenesis on three different CRMs discovered using two different computational approaches. For all three CRMs studied, we show that one or both of the following holds: (1) the majority of TFBSs used to construct the search model make little or no necessary contribution to activity of the CRM, or (2) the transcription factors assumed to be acting at the constituent TFBSs are not in fact involved in regulating activity of the CRM. These results raise questions about the effectiveness and accuracy of using defined sets of conserved TFBSs for CRM discovery and highlight the importance of empirical validation even when results are consistent with initial assumptions.

## Results

We previously conducted a computational search for CRMs directing gene expression in the *Drosophila *embryo by searching for local co-occurrence of specific TFBSs [11]. Specifically, we took as a model a CRM of the gene *even skipped (eve) *for which we had defined five positively-acting transcription factors (dTcf, Mad, Pnt, Tin, and Twi) that direct *eve *expression to the embryonic dorsal mesoderm (the Eve MHE) [15] and searched the genome for regions in which binding sites for all five factors occurred within 500 bp of one another. We then filtered the 647 found regions to select those for which there were at least two instances of the TFBS for each factor (with the exception of dTcf, for which we allowed one or more instances); this "selected subset" comprised 33 putative new CRMs. Of seven sequences tested in vivo for CRM activity in the embryo, we found that one (the Hbr DME) was able to drive gene expression in a pattern closely resembling that of the model Eve MHE enhancer, but the other six failed to function as CRMs completely. Despite the low overall success rate for our computational search (1/7 or 14%), these results demonstrated that the TFBS co-occurrence model we had defined was capable of enabling discovery of similar-acting CRMs [11].

**Figure 1:**
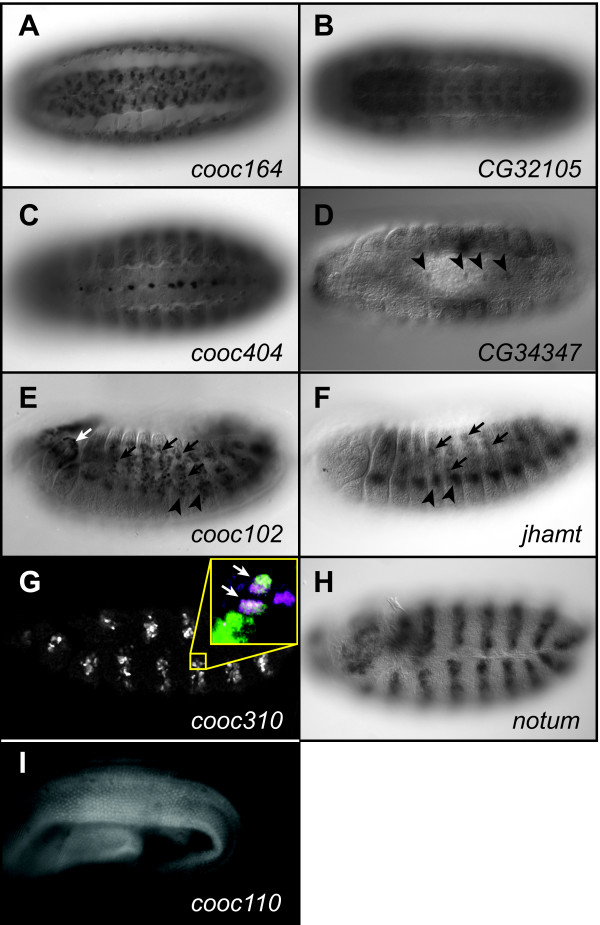
**Successful prediction of new CRMs**. Reporter gene expression is shown in the left-hand panels (anti-GFP: A, C, E, G; GFP fluorescence, I), in situ hybridization to mRNA of the assigned target gene in the right-hand panels (B, D, F, H). Embryos are oriented anterior to the left and ventral side up (A-D) or laterally with dorsal to the top (G-H). (A) *cooc164 *drives reporter gene expression primarily in the ventral nerve cord in a pattern similar to that of target gene *CG32105 *(B). (C) *cooc404 *drives gene expression in the midline of the ventral nerve cord. (D) *CG34347*, putative target gene for *cooc404*, is expressed in the same midline cells (arrowheads). (E) Reporter gene expression from *cooc102 *can be observed throughout the mesoderm (black arrows, arrowheads). Expression in the visceral mesoderm (not shown) and anterior segments (white arrows) is not observed for the assigned target gene *jhamt *(F). Arrows and arrowheads in panel F mark somatic mesodermal cells corresponding to those similarly marked in panel E. (G) *cooc310 *reporter gene expression is observed in segmentally repeated stripes, primarily in the embryonic ectoderm. *Inset *shows cells in the mesoderm co-labeled (white cells, marked with arrows) for GFP (green) and the mesodermal marker Mef2 (magenta). (H) Corresponding stripes of expression are seen for target gene *notum*. (I) *cooc110 *drives gene expression in pupal tissues including the wing.

As a follow-up to this study, we attempted to determine whether evolutionary conservation could be used as a filter to improve the accuracy of our CRM prediction. We aligned the sequences of the 647 putative CRMs from our initial search--those having one or more instance of each of the five different TFBSs--with the genome of *Drosophila pseudoobscura *(at the time the study was initiated, in 2002, the only other sequenced *Drosophila *genome) and selected sequences with strong blocks of sequence conservation and in which most of the TFBSs used in our predictions were conserved (see Methods). We selected six of these putative CRMs to test in vivo by reporter gene assay in transgenic flies (Table 1). Remarkably, five of the six newly selected sequences were functional CRMs (Figure 1, Table 1). *cooc164 *drives reporter gene expression in the embryonic ventral nerve cord and the amnioserosa (Figure 1A and data not shown) and lies in the intergenic region between genes *CG4328 *and *CG32105*. Based on locations of putative insulator sequences [16] and similarity in expression pattern (Figure 1B), we assigned *CG32105 *as the target of this CRM. *cooc404 *lies within an intron of *CG34347 *and drives gene expression in cells along the midline of the embryonic ventral nerve cord--consistent with expression of *CG34347*--and in the amnioserosa (Figure 1C, D). A shorter version of this CRM sequence also drove expression in the nerve cord but in a broader pattern than *CG34347*, presumably due to lack of repressor sequences contained within the larger construct (data not shown). For both of the above two CRMs, amnioserosa expression may be ectopic as the target genes do not show significant expression in this tissue (data not shown). The *cooc102 *reporter gene is expressed in somatic and visceral muscle from embryonic stage 12 onward (Figure 1E). The CRM lies approximately 1 kb upstream of *jhamt*, which is expressed in somatic muscle (as well as other tissues) although not in visceral muscle (Figure 1F; see also [17]). Due to the overlap in somatic muscle expression and annotated insulator sequences, we assign *cooc102 *to *jhamt *and consider the visceral muscle expression as ectopic. *cooc310 *drives reporter gene expression in segmentally repeated stripes in the germband extended embryo, consistent with the expression of *notum*, which lies approximately 2 kb downstream of the CRM (Figure 1G, H). This expression is primarily ectodermal but a few subsets of mesodermal cells also express the reporter gene (Figure 1G inset and data not shown). *cooc110 *lies within an intron of *beat-IIIa *and is expressed beginning at mid-to-late pupal stages most strongly in the wing but also in the legs, eyes, and other tissues (Figure 1I and data not shown).

**Table 1:** CRM predictions tested in this study

CRM name	coordinates (r5/dm3)	reporter activity	putative target gene	primary expression pattern
*CG32105_cooc164*	chr3L:12306977-12307990	+	*CG32105*	ventral nerve cord, amnioserosa
*CG34347_cooc404*	chr3R:27173861-27175568	+	*CG34347*	ventral nerve cord (midline*), amnioserosa
*cooc110*	chr2L:17140480-17141559	+	*beat-IIIa*	pupal wing, eye, leg and other tissues
*cooc437*	chr3R:19617439-19618229	-	*n/a*	none
*jhamt_cooc102*	chr2L:16364204-16365055	+	*jhamt*	somatic and visceral muscle from stage 12 onward
*notum_cooc310*	chr3L:16013542-16014711	+	*notum*	segmentally repeated stripes, mostly ectodermal with limited mesoderm expression in dorsal regions and in the anal ring

*a shorter version of cooc404 also had ventral nerve cord activity but ectopic relative to CG34347 expression (data not shown)

Contrary to expectation from our search for dorsal mesoderm regulators, none of these five new CRMs drive a gene expression pattern similar to that of the Eve MHE and Hbr DME and only two (*jhamt_cooc102, notum_cooc310*) of the new sequences direct any gene expression in the mesoderm at all. We also note that *cooc110 *would have been scored as "negative" for CRM activity in our previous study, as the pupal tissues where this CRM functions were not assayed in our original validation experiments. Nevertheless, these results represent a significant improvement over the prior round of CRM prediction, which used the same search results but without consideration of sequence conservation.

In order to better understand the factors leading to the substantial increase in predictive success we observed, we divided our set of 13 predicted CRMs into two groups representing the six positive and seven negative predictions. We used four methods to assess the degree of conservation for each sequence (Table 2): the original AVID-based alignments; local realignment using DIALIGN [18]; and 15-way PhastCons scores [19] obtained via the UCSC Genome Browser [20]. The latter were calculated both as the average score over the whole tested CRM sequence and using the "peakPhastCons" method shown by Su et al. [21] to have good discriminatory power in an assessment of CRM discovery methods for *Drosophila*. Although by each measure the positive predictions show higher conservation than the negative predictions, the differences are small and for the most part not statistically significant. This is consistent with the results both of smaller similar studies of CRM prediction [9] and of our large-scale analysis of close to 300 CRMs [22]. The exception is with the peakPhastCons method at larger window sizes, as previously shown [21]. Note however that many of the CRMs from the positive set have scores in the range of those in the negative set (and vice versa), demonstrating that while significant in the aggregate, the peakPhastCons method will give many false positives and false negatives when used to predict individual CRMs. We also assessed the conservation of individual TFBSs rather than of the entire predicted CRM sequences. Previous work by Berman et al. [9] showed that binding site conservation was a good discriminator between true and false positive predicted CRMs. Contrary to these results, however, we found that while the positive set on average has a higher degree of TFBS conservation than the negative set, once again these differences are not statistically significant (Table 2).

**Table 2:** CRM activity and conservation

CRM^a^	activity	AVID	AVID+ DIALIGN	phastCons (average)	peakPhastCons (100 bp)	peakPhastCons (200 bp)	peakPhastCons (500 bp)	TFBS presence^b^	TFBS mismatches^c^
**cooc164**	+	0.6002	0.7710	0.5532	0.6651	0.6337	0.6431	0.8	0.21
**cooc102**	+	0.6345	0.8230	0.4246	0.4836	0.5038	0.5325	0.8	0.15
DME2	+	0.6694	0.7860	0.4472	0.5416	0.5296	0.5133	0.8	0.19
**cooc310**	+	0.6766	0.5250	0.6649	0.7616	0.7459	0.8099	0.8	0.26
**cooc404**	+	0.6874	0.7530	0.6466	0.6718	0.6740	0.6778	0.8	0.29
**cooc110**	+	0.6867	0.7150	0.5072	0.5808	0.5767	0.5930	0.8	0.34
	**+ Average**	**0.6591**	**0.7288**	**0.5406**	**0.6174**	**0.6106**	**0.6283**	**0.8**	**0.24**
	**+ StdDev**	**0.0348**	**0.1061**	**0.1001**	**0.1010**	**0.0916**	**0.1089**	**0**	**0.07**
									
DME31	-	0.5037	0.5010	0.4509	0.6249	0.4786	0.4628	0.6	0.38
DME30	-	0.5597	0.5840	0.5403	0.6183	0.5900	0.5292	0.4	0.35
DME7	-	0.5819	0.5350	0.5822	0.5609	0.5103	0.4809	0.6	0.20
DME3	-	0.6095	0.6920	0.5168	0.4784	0.4826	(<500 bp)	0.8	0.22
DME25	-	0.7401	0.8830	0.4480	0.5255	0.4964	0.4640	1	0.14
**cooc437**	-	0.6430	0.7840	0.5899	0.5970	0.5971	0.6145	0.6	0.12
DME4	-	nd	nd	0.5025	0.6021	0.5307	0.5200	nd	nd
	**- Average**	**0.6063**	**0.6632**	**0.5187**	**0.5724**	**0.5265**	**0.5119**	**0.67**	**0.23**
	**- StdDev**	**0.0807**	**0.1502**	**0.0569**	**0.0540**	**0.0490**	**0.0575**	**0.21**	**0.11**
									
p value (Wilcoxon one-sided)		0.08983	0.2424	0.4726	0.2226	0.0507	**0.0206**	0.06287	0.5909
								
with DME25 switched		**0.008838**	0.05303	0.6859	0.4178	0.1474	0.1010	**0.005269**	0.3194

^a ^**Bold **type indicates CRMs tested for activity in this paper; others are from [11].

^b ^TFBS presence was scored as number of sites present in *D. pse*. divided by number of sites in *D. mel*.

^c^TFBS mismatches were scored as the fraction of unaligned nucleotides in each *D. pse*. vs. aligned *D. mel*. site averaged over all of the *D. mel *TFBSs for the CRM region (e.g., a fully conserved site = 0, a completely unaligned site = 1, average for the CRM if these were the only two sites = 0.5).

The modest apparent contributions of both CRM-wide and TFBS-specific conservation were surprising, given the dramatic increase in prediction success we attained when using conservation as a guide in selecting sequences to validate (83% with CRM activity vs. the previous 14% with CRM activity). We reasoned that there might be several explanations for this result. One, we recognized that the small overall sample size makes these statistics very sensitive to the effects of individual sequences such that a single misclassified predicted element could have a dramatic outcome. For instance, were the element DME25 to be moved from the negative to the positive set, several of our measures of conservation would then show a significant difference (Table 2, row "DME25 switched"). This is an important concern, as we have already seen how such an error could have occurred with respect to *cooc110*, which regulates gene expression in a tissue not assayed in the 2002 study. Thorough testing of a significantly larger number of predictions would be necessary to rule out this possibility. Two, we may not have identified the appropriate method for scoring conservation. For instance, the peakPhastCons method with a 500 bp window size shows better performance than the other measures, although it would still have led to many incorrect predictions. Other, superior methods may still be found. Three, our measures of TFBS conservation might be inaccurate, given our incomplete knowledge of each TF's binding motif and the limitations of methods for identifying binding sites. That is, we may be incorrectly considering a pair of sites to be non-conserved due to sequence differences that in reality allow equally effective binding, or conversely, attributing conservation to similar sites that do not, in fact, both mediate binding of the correct TF. Finally, we may be erring in which TFBSs to consider. Because our CRM search was predicated on the co-occurrence of five individual TFBSs, our assessment of conservation considered all five motifs. However, we note that none of the new CRMs chosen based on sequence conservation regulate gene expression in the precise dorsal mesoderm pattern seen for the Eve MHE and Hbr DME. Therefore, these CRMs may not have the requirement for binding all five TFs stipulated by our model. In that case, considering the conservation of all five sets of TFBSs would be detrimental, adding the noise of potentially non-conserved non-important sites to the signal of conserved, necessary sites. This interpretation is consistent with the findings of Philippakis et al. [13], who in an independent search using the same five TFBSs found that not all sites contributed equally to the set of predicted CRMs and that jointly considering all five TFBSs led to a worse result than considering subsets of the TFBSs.

**Figure 2:**
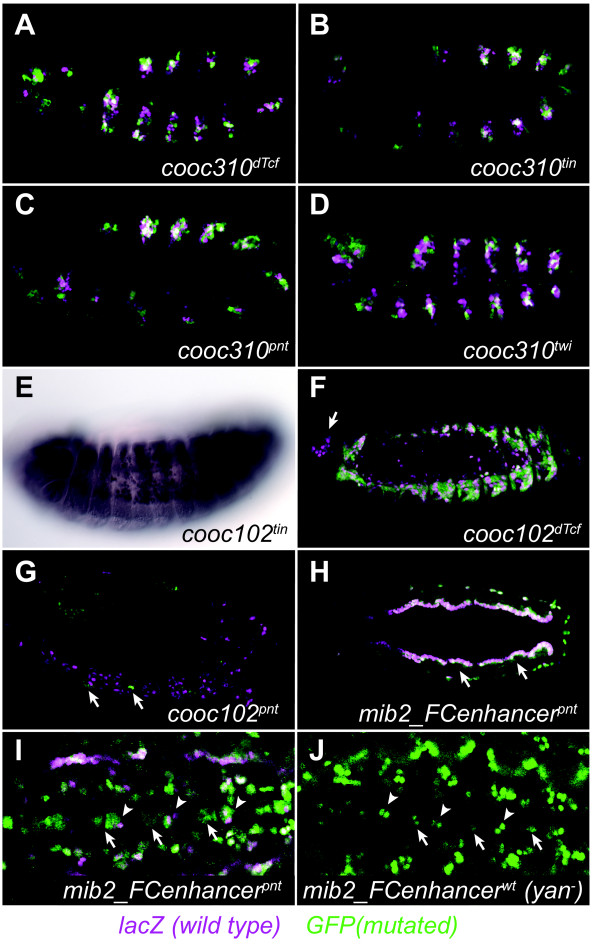
**Effects of binding site mutagenesis on reporter gene expression**. Flies with the wild-type *cooc310*, *cooc102*, or *mib2_Fcenhancer *driving nuclear lacZ were crossed to flies with mutated versions of the CRMs driving cytoplasmic GFP and the resulting embryos double-stained for both reporters (lacZ, magenta; GFP, green; overlap, white). Areas of direct overlap are limited due to the nuclear vs. cytoplasmic expression of the two reporters but coincident expression can readily be observed. Embryos are oriented anterior to the left and dorsal to the top, except for panel H, which is a dorsal view. (A-D) Mutagenesis of dTcf, Tin, Pnt, or Twi predicted binding sites in the *notum_cooc310 *CRM have no discernable effect on CRM activity. (E) Mutation of the Tin site in CRM *jhamt_cooc102 *causes a quantitative reduction in reporter gene expression but has no effect on expression pattern (compare with Fig. 1E). (F) An intact dTcf site is required for expression mediated by *jhamt_cooc102 *in the anterior (arrow) but has no effect on the remainder of the reporter gene expression. (G) Mutation of the *jhamt_cooc102 *Pnt site leads to a near-total loss of reporter gene expression. Arrows indicate a few remaining GFP-positive cells. (H) Putative Pnt binding sites in the *mib2_FCenhancer *CRM lead to an expansion of reporter gene expression throughout the trunk visceral mesoderm when mutated (arrows) and to additional cells with reporter gene expression in the ventral midline (panel I). Somatic mesoderm cells around the periphery of the pictured embryo express both reporters. (I) Close-up view of the stage 11 ventral midline showing additional cells expressing the mutated *mib2_FCenhancer *reporter gene (arrows). Arrowheads mark cells which also have expression driven by the wild-type enhancer. (J) In a *yan^- ^*background (*yan^XE18^*), additional cells express the wild-type *mib2_FCenhancer *reporter gene (arrows). The smaller apparent size of these cells compared to the similar arrow-marked cells in panel I is mainly due to the cytoplasmic vs. nuclear nature of the two reporter genes, although we cannot fully rule out additional ectopic expression using the mutated enhancer. Arrowheads indicate the same wild-type *mib2_FCenhancer*-expressing cells marked with arrowheads in panel I.

In an attempt to shed additional light on this latter possibility, we undertook extensive mutagenesis of two of the newly-identified CRMs, *notum_cooc310 *and *jhamt_cooc102*. We mutated all of the dTcf, Tin, or Pnt motifs used for prediction of each CRM, as well as the Twi motif used for prediction in *notum_cooc310*, and assayed the ability of the mutant CRMs to drive reporter gene expression in transgenic embryos (Figure 2 and Additional File 1, Fig. S1). Surprisingly, we found that most of the mutations had little or no demonstrable effect on reporter gene expression. Activity of *notum_cooc310 *was essentially unchanged in all four mutated CRMs (Figure 2A-D). For *jhamt_cooc102*, Tin site mutation caused a quantitative reduction in reporter gene activity but no change in expression pattern (Figure 2E). Elimination of the dTcf site caused a loss of reporter gene expression in a group of cells in the anterior of the embryo, but the majority of the expression pattern was unaffected (Figure 2F). Knockout of the single Pnt binding site in *jhamt_cooc102 *caused the only extensive phenotype we observed, an almost complete loss of reporter gene expression in the embryo (Figure 2G). Surprisingly, the Pnt site was the least conserved of the analyzed sites in *jhamt_cooc102*, with no appreciable conservation in *D. pseudoobscura*. Moreover, *jhamt_cooc102 *reporter gene expression appears to be normal in a *pnt *mutant background (data not shown), suggesting that in designing the Pnt-site knockout construct we may have unknowingly affected a binding site for a different TF that binds a similar or overlapping sequence.

Despite the fact that our search led to successful discovery of new CRMs, the above results suggest that most of the TFBSs on which the search was based have little or no requirement with respect to activity of the identified CRMs. Although the details vary, the basic strategy used in our search--identification of clusters of specific binding sites filtered by evolutionary sequence conservation--has been one of the main approaches taken to computational CRM discovery [3,4]. We therefore wondered if it might also be the case for other instances of successful CRM discovery that the TFBSs used as input to the search would not turn out to be major functional components of the identified CRMs. To test this, we examined the role of Pnt binding sites in the *mib2 *mesodermal enhancer (REDfly: *mib2_FCenhancer*) discovered in the computational search of Philippakis et al. [13]. This search used a sophisticated computational algorithm, ModuleFinder, based on TFBS motifs and sequence conservation, to identify sequences containing binding sites for Pnt, Twi, and Tin (a subset of the sites used in our own original search). Subsequent filtering based on microarray-derived gene expression profiles, based on a model in which these three TFs serve as activators of gene expression, was used to choose candidates for in vivo testing. Importantly, the Philippakis et al. model was explicitly optimized for relevant binding sites (those contributing signal, not noise, to the search). In addition, not only did the *mib2 *CRM score high in this search, but *mib2 *was among a subset of genes determined to be highly upregulated in *pnt *gain-of-function microarray experiments [23]. Contrary to expectation, mutation of the five putative Pnt binding sites identified by the ModuleFinder search, plus two additional sites that were also potential matches to the Pnt binding motif (Additional File 1, Fig. S1), caused an expansion, rather than a reduction, in reporter gene expression (Figure 2H). Expression of the wild-type *mib2 *CRM reporter in the visceral mesoderm is restricted to the muscle founder cell population, but with the mutated CRM it expands throughout the visceral mesoderm. This expansion is not seen in *pnt *null mutant embryos (using either the wild type reporter construct or direct detection of *mib2 *expression by in situ hybridization; data not shown). Neither is it seen in embryos mutant for the repressor Yan (FlyBase: *aop*), which is known to bind the same sequences as Pnt (data not shown). Similar to what we observed for *jhamt_cooc102*, therefore, it is possible that when constructing our Pnt-site mutations we inadvertently disrupted sites for a different TF. We did observe a gain of reporter gene activity in a subset of cells in the ventral midline, at least part of which appears to result from loss of repression mediated by Yan (Figure 2I, J). These results show that there is in fact a functional role for what were described as Pnt binding sites in the *mib2 *CRM. However, this role appears to be a minor one relative to the presumed primary activating role implicit in the model used to construct the computational search. Thus for a CRM found by the ModuleFinder algorithm [13], just as for the CRMs identified through our own TFBS co-occurrence based search, TFBSs that formed the basis of a successful prediction may not play a large role in mediating activity of the discovered CRM. Furthermore, the TF believed to be acting via those TFBSs was incorrectly attributed.

## Discussion

Clustering of TFBSs, singly or in combination, has frequently been used as a method for computational CRM discovery. While false-positive prediction rates remain high for many studies, when CRMs are positively identified by these methods, it is assumed that the TFBSs that were used as input to the prediction algorithm are important functional regulators of the CRM's activity, as are the transcription factors that bind to these sites. Although in some cases this has been shown to be the case [e.g. [11]], more often than not the assumption is allowed to rest unchallenged. Here, we show through extensive empirical testing in vivo that many of these specific TFBSs, and/or their assumed cognate transcription factors, appear to be relatively or completely unimportant for CRM activity. This leads to the somewhat paradoxical result that although consideration of the putative TFBSs led to successful CRM discovery, the sites in many cases do not appear to be functional CRM constituents. What explains this finding?

One relatively trivial answer would be if most randomly selected sequences could act as CRMs in a reporter gene assay. However, we discount this possibility based on our own initial search results in which only one out of seven tested sequences showed regulatory activity [11]; on similar results by others (see e.g. Table S8 in [24], and ref. [3]); and on considerable amounts of anecdotal data suggesting that despite the relative compactness of the *D. melanogaster *genome, many if not most sequences will test negative. A further possible explanation would be if most *conserved *sequences acted as CRMs. Again, however, it is hard to credit this scenario given the close similarities in extent of conservation seen with true- versus false-positive predictions by both ourselves and others (e.g., Table 2 and [9]).

It could also be that the input TFBSs are indeed functional, and that our assay failed to confirm this. For instance, there may be subtle quantitative effects. Or, we may have failed to identify additional binding sites, in which case the sites we mutated may be functional but not essential, due to the presence of other, redundant sites for the same TFs. Ruling out such possibilities will require extensive testing of each individual CRM and TFBS. We do note that chromatin immunoprecipitation experiments have failed to find evidence of Tin or Twi binding to either the *notum_cooc310 *or the *jhamt_cooc102 *loci [25,26], consistent with the lack of phenotype we observed upon TFBS mutation. We also point out that we only chose to mutate the sites identified in our original CRM-prediction search, using motif definitions and methods available at that time. Although searching using updated motif data and/or different search algorithms and parameters does suggest potential additional sites for some of the TFs (data not shown), mutagenizing these sites would prohibit the key assessment we make here: given successful CRM prediction using a specific set of identified TFBSs, are those specific sites and/or their cognate TFs necessary for CRM function? In effect, taking into consideration TFBSs not part of the original CRM prediction would be little different from a situation in which we would take the discovered CRM and run a post-hoc analysis that finds a previously unrecognized site for a TF not part of the search model. While we could later show such a TFBS to be functional, we could not claim it as a basis for discovering the CRM in the first place.

We favor the view that identification of conserved TFBSs can aid in CRM discovery, but suggest that great care must be taken in imputing functional roles to these sites in light of the high false positive rates inherent in TFBS prediction. The ability of confirmed *in vivo *TF binding to enable CRM discovery has been clearly demonstrated by ChIP-based studies [e.g. [25-28] (although even *in vivo *binding does not necessarily connote function [29], and computational predictions can sometimes outperform ChIP-based ones [30]). However, in general computational methods are poor at distinguishing functional from non-functional TFBSs. The explanation best supported by our data is that it is necessary to identify *functional *rather than just *putative *TFBSs. Many of the putative TFBSs we tested turned out to be nonfunctional, and we suggest that these added enough noise to our search results that we could not reliably demonstrate the utility of the functional conserved sites in driving success of the search. In two of our three tested CRMs, we did observe that some of the considered TFBSs were functional. Although their roles were relatively minor with respect to the complete expression patterns regulated by the CRMs, we posit that, as bona fide TFBSs, they were sufficient to aid in CRM discovery. On the other hand, the TFBS with the most essential role, the Pnt binding site in *jhamt_cooc102*, was not strongly conserved and therefore not likely to have contributed significantly to the improvement in search success rates achieved by considering TFBS conservation.

## Conclusions

Simultaneous searching for TFBSs for several TFs has often been proposed as one means to reduce false-positive rates for purposes of CRM discovery, and a number of studies, including the present one, support its basic effectiveness. However, our results raise important questions about interpreting successful predictions using such an approach: many of the different sites we identified, even when co-clustered, appear to be non-functional. This is true even though the combinations of sites that served as input into the searches were selected as part of the "transcriptional code" for the genes being sought. For example, although *jhamt_cooc102 *drives gene expression in the mesoderm, both our results and ChIP data from the Furlong lab [25,26] suggest that this expression is mediated by neither Tin nor Twi, two well-known mesoderm-specific TFs (although *jhamt_cooc102 *does appear to be bound by Mef2, another mesoderm-specific TF [31]). Similarly, while the search for *mib2_FCenhancer *was focused on joint occurrence of Tin + Twi + Pnt, not only does mutagenesis of the putative Pnt sites have a de-repressive rather than negative effect on the CRM, but the CRM is fully functional even in the complete absence of the *pnt *gene. Thus our ability to understand so-called transcriptional codes and make use of them in a prospective manner may be less than we believe. Considering the combinatorics of TF binding, rather than being important in order to increase the probability of zeroing in on the right assortment of binding sites, may simply increase the probability of finding *some *functional sites by virtue of increasing the total number of sites being predicted.

Recent studies in yeast raise similar concerns about assuming functional roles for TFs based on the presence and conservation of putative TFBS sequences [32]. Despite the simpler *S. cerivisiae *gene structure in which most regulatory interactions take place within the roughly one-half kilobase of sequence upstream of the transcription start site, many conserved predicted TFBSs were shown to be non-functional, and many functional sites not well conserved. These data, taken together with the results presented here, underscore the importance of conducting thorough empirical validation of computational predictions even when results seem to be in line with expectation.

## Methods

### Prediction of new CRMs

CRM predictions are described in [11]. Alignments of the 647 elements identified in that search to *D. pseudoobscura*, based on the AVID alignment software [33], were obtained from the Berkeley Drosophila Genome Project (pipeline version 8 July 2003, http://pipeline.lbl.gov/pseudo/). Elements that ranked high in both overall and TFBS conservation were chosen for in vivo testing. Sequences and additional information for the tested CRMs are provided in Table 1 and are available via the REDfly database (http://redfly.ccr.buffalo.edu; [34]).

### Sequence alignments

AVID alignments were obtained as described above. The value reported in Table 2 is the percentage of conserved nucleotides within the sequence of the in vivo tested CRM. The aligned regions were then extended 700 bp in each direction from the ends of the *D. melanogaster *CRM sequences, and the resulting *D. melanogaster *and *D. pseudoobscura *sequences were realigned using DIALIGN [18]. Ends of the aligned regions were defined as the borders of the first and last conserved ungapped blocks, respectively, that contained one of the TFBSs defined for the CRM, and the overall percentage of aligned nucleotides contained within conserved blocks reported. PhastCons scores [19] were obtained from the UCSC Genome Browser [20] based on the tested CRM sequences. The "peakPhastCons" method was performed as described by [21] with window sizes of 100, 200, and 500 bp and a cutoff of 0.13, except that only the sequences of the tested CRMs were analyzed (i.e., the first scored window began at the first basepair of the CRM and the last window ended at the final basepair of the CRM).

### Reporter constructs, transgenesis, and mutagenesis

Reporter constructs, site-directed mutagenesis, fly transgenesis, and analysis were all as previously described [11] except that for some constructs, GFP was used as the reporter instead of lacZ. Mutagenized sites are shown in Fig. S1. Ability of the specified basepair changes to abrogate binding of the relevant TFs has been shown previously [15]. DIC microscopy was performed using a Zeiss Axioskop 2 microscope with a Retiga-EXi camera (Qimaging) and Openlab software (PerkinElmer). Fluorescent images were acquired using a Leica SP2 confocal microscope. All images were color corrected and contrast adjusted in Adobe photoshop.

## Competing interests

The authors declare that they have no competing interests.

## Authors' contributions

QZ and MSH cloned and analyzed new CRMs. ERB and YZ made and analyzed mutated CRM constructs. MSH designed the study and wrote the manuscript. All authors read and approved the final manuscript.

## Supplementary Material

Additional file 1 **The following additional data are available with the online version of this paper: Additional data file 1 is a PDF document containing Fig. S1**. Click here for file
