# Supplementary material for: Erroneous attribution of relevant transcription factor binding sites despite successful prediction of cis-regulatory modules
Source: BMC Genomics. 2011 Nov 25;12:578. doi: 10.1186/1471-2164-12-578 (PMC3235160; doi:10.1186/1471-2164-12-578)
Supplement: Additional file 1 — The following additional data are available with the online version of this paper: Additional data file 1 is a PDF document containing Fig. S1. [file 1471-2164-12-578-S1.PDF]

Supplementary Figure S1: CRM mutagenesis. Panels A-C provide a graphical summary of the CRMs, their binding sites, and the results of mutagenesis. Browser snapshots from the UCSC Genome Browser depict flanking genes and PhastCons scores for each CRM. CRMs are indicated by the heavy bar marked as "your sequence from Blat search." The expanded view below each browser snapshot shows the TFBSs, color coded as follows: ETS (Pnt), red; Tin, blue; Twi, green; dTcf, orange. "X's" represent mutated sites.

Panels D-F provide the nucleotide sequences of the CRMs. The predicted binding sites are underlined. Site-specific mutations are depicted below the mutated sequence, color-coded as in A-C. For each mutated CRM, all of the indicated TFBSs for a given TF were mutated in the same construct.

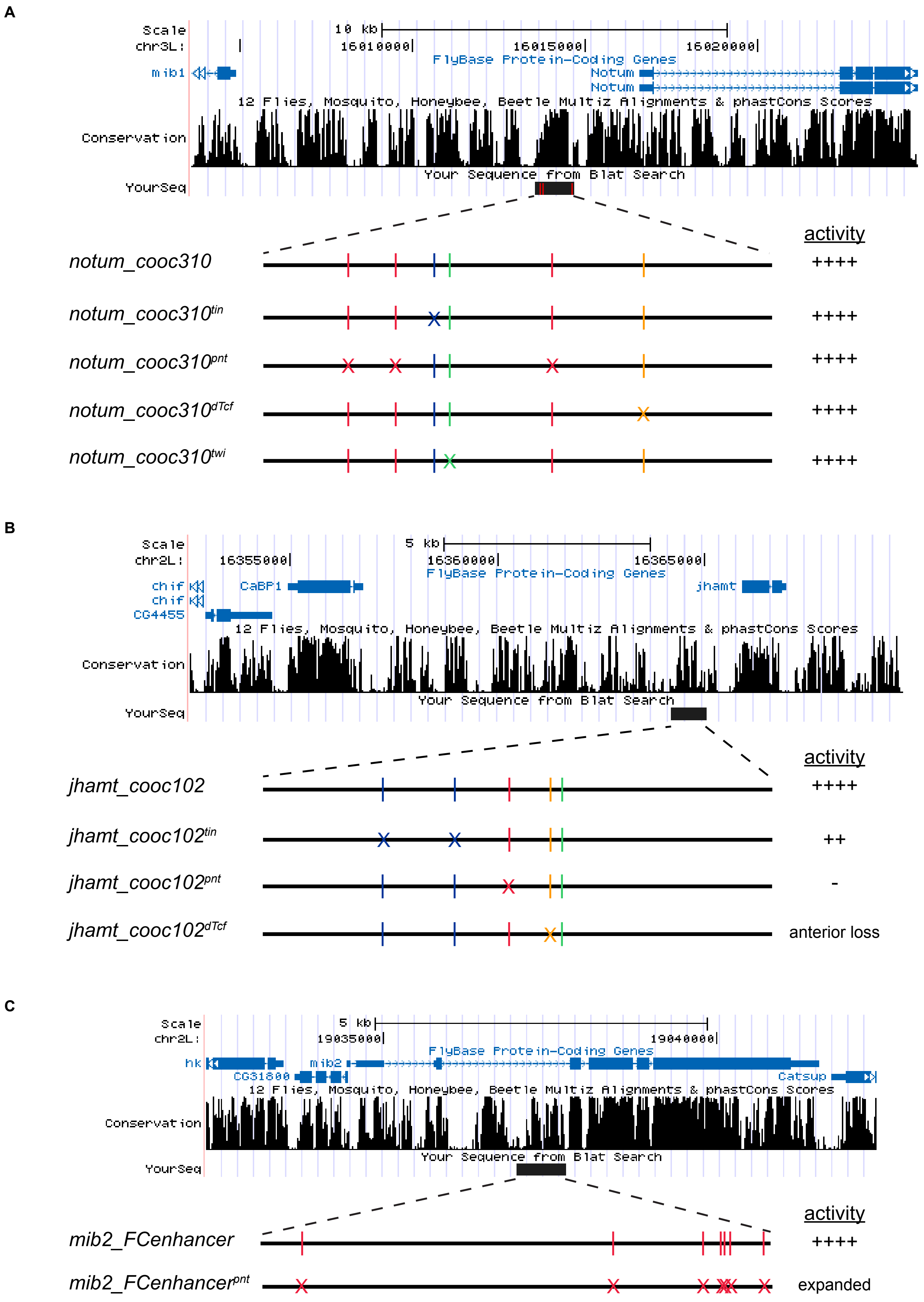
A
Scale
chr3L:
10 kb
16010000
16015000
16020000
FlyBase Protein-Coding Genes
mib1
Notum
Notum
12 Flies, Mosquito, Honeybee, Beetle Multiz Alignments & phastCons Scores
Conservation
Your Sequence from Blat Search
YourSeq
activity
notum_cooc310
++++
notum_cooc310^tin
++++
notum_cooc310^pnt
++++
notum_cooc310^dTcf
++++
notum_cooc310^twi
++++
B
Scale
chr2L:
5 kb
16355000
16360000
16365000
FlyBase Protein-Coding Genes
chif
chif
CG4455
CaBP1
jhamt
12 Flies, Mosquito, Honeybee, Beetle Multiz Alignments & phastCons Scores
Conservation
Your Sequence from Blat Search
YourSeq
activity
jhamt_cooc102
++++
jhamt_cooc102^tin
++
jhamt_cooc102^pnt
-
jhamt_cooc102^dTcf
anterior loss
C
Scale
chr2L:
5 kb
19035000
19040000
FlyBase Protein-Coding Genes
hk
mib2
CG31600
Catsup
12 Flies, Mosquito, Honeybee, Beetle Multiz Alignments & phastCons Scores
Conservation
Your Sequence from Blat Search
YourSeq
activity
mib2_FCenhancer
++++
mib2_FCenhancer^pnt
expanded

ETS: RED
TIN: BLUE
TWI: GREEN
TCF: ORANGE

D: notum_cooc310: **3L:16013542..16014711**

TTCAGTAAAATCCTTTAAAATTTCCATAAAGCACCTGGTGACCATGCAACATCACTGACCTCCGTCAATCGAGAGC

AATGCAATTATGAACCCCGGCTGGAAGAAATTAAAGATGCTCGCTCCGCCGGCTGATGGATGAGAAATGCTGCTTC

CGACTGGAGATTGGATGCTGGATTCTGGATGCTGGAGCCTCGGCGCACCTGTTGTGTTTCCCACAGCGAGCGGACG
CTTA

CTTTGGTTTTTCGCAAAATAAAGATGAAAAAACACCGGAGAACAGTTGTGCAGCGGATCCTGAAATCGGGCAAAAG
CTGA

GATCAAAGGACGTGACCGTGACTGGGCCGAACCGATAATAAAGTTGTGCATTAAATGAAATTTTAATTGCTTATCG
TGAG

GCGACCGGCGATCCGGGAGCCCATCTGTTGGGATTAGAGCGGCCGAATGGCCGACTCGATTGATGTCAAGCGATCG
CTCC

GTCGGTCGGCGCTTTGAAGATTATGCCCAAAGTTATGAGGTTGCGGTAGAGGCTCTGTATTCCAGGTGCTCCGTTC

TCCAGGTGATCGGATCGGCCTATGCGAAATTATATAGCTCAGCAGCGTTGGTAGCGATGTAGCAGCTTTAACTACG

CAGTGCCTGTCGCTTCCTCGCCGTCCTCTTTCTCGTCATCGTCATCGTCATCATCCTCATCATCATCATCATCATC
TAGG

ATCATCATCATCACCGTCATCATCTTCCGCTGGGGCATCGCATCAGCTGGCGCTGCTGCTTGTTGCTTGCTGCTTG

CTATTTGCTGCATGCGGCAGAAGATGAAACGCGGCGGCACAACTTTGATGCGGCCAAGATCAAAGCCGCCGATTGT
TCGTCC

TTACATAATAGTGGCCTTTGAAACGGAACAATAGAATGCCAGGATCAAAGACACCGACCAGTTTACCCGGTTAGTG

CGCTTAAGCGAAATGAATGTCCATGTCCGTCCGCCCGTGATCTGTTTTTCCTGGCCCCACACCGCAAGAAAATGTA

TGCTTTTTGGGATCAATAAAAACCATTTGAGTATGCTTTATCAAATCAACAAATACTAACTATCTAGTTTTTCAGT

AAGTACTTGTTTTTAACCAAGTCAGCTAAAATGTTTAGTTAATTCATTTTGGACAAAGGTTTTGTTGCAGTGTAGT

AAAAGTAGCACACCTTGCTGGGGTTGGAA 1169

E: jhamt_cooc102: **2L:16364204..16365055**

ATGTTCCAGCTTATGCCTTAAGACCACACTTATCTAAATTAATCTGCACTGTGTCATCTCGCAGAATAATTGATTT

ATGTGCTATAACTTCATTAAAATACTCAAAGCCAAGAAAAGCAAAACCCTCAAACTCCCAAATGAATCGAAATGCT

GAGGCTCTCTGTTTGGTTCGTTTCGGTTTGGTTCGTCCGATTTAATTGATTTCCGTGGCTCTGTGACAGCTCGAGC
TGAG

CAATCGACAGCTCTCGGTAATTTCGAGCCGAATAACCCTTTTGCTGACTTTTGCCTGTTTTACCAAAAAGAGTCTG

CGACAAATGCAATTAACCAGCCGTAACGCCCACACACATTAAGCCAGAAACAGAATCCGAGCGAGGGCTTTTGATG
CTCA

TCACTTTGGCAAATGAATTTATATGGATGTGATGTGCCCGAGTGTTGGAGAATGCAGGATACGAACGTGAGTGGGA
CCTA

GTTCATAACTCCCAGGGACATCAAACGGTCGTTCTGCTCATATGTGAAATATTTCAAATAACATGAGCTTCGCTTT
TCGTCG

CTGCCAGCAATTTCTGCCAGCAATTACACAAATGTAATTTGCAGCTAAATGGAATGTTTTTAATGAATTTATATTT

CCCGCAGCATCGAAGAGTGGGTCCAAAATAATTTCCCCTTTCTATTTCTATTGTTCGAACATCAAATTATTGGTAT

ATATTTATCCAAAAATAGATTAGATTTCGTTTTCGAACCAATCTTAGGGTAGGTCATCGAACTGACTTCACTCGTT

TTAATAGTTTCTTGAGTTGAATGCACCTCCAACACTTTTTTCTTTACTTCTGCACATTCGGGTTCATCGAAAATTA

GGATCGTTTTTGGGC 851

F: mib2_FCenhancer: **2L:19036995..19037741**

CCAAGAGAGAACATTTCTTCAGATACACTTCAAAAACAATTTGAATCATTTT<u>GTGGAAGT</u>TAAACCAATCACCAAA
CG

GCCTAATTTGATAATAAACGCAGTAATGTTTAAGCCTATTGAAGGATCCTATTAAAGCAGCTGCACACGTTTAGGA

TTAGGTCATGGTATGTGTTCCCCCCTCCCCAAGTCGCCCTATTCCAGCCTGTGCAGCGAATGCATATGCACATGTC

AGGCGCTAGGGCGCGTGCCATTTTGTCTCCAACCTTTTTTTGAGTGTTTATTTTTAGTTACACGGGTGGGGATCGC

GAGCAGTTACACAGCTCCAGCCGGCCTGATATTTGGGGACGATGTGGAGAGTTTATTTTTGGACCTTGGTATTCCT

CCGACTGGGGTGTGTTGAAAATAGACATAAACAAATTTTCAGATGGGAGCCCAGCCATCGGTGCTCCCATTCCTCC

CCAGGCCAACATCAAATGTCACCCTCTAGAGAACGATTCC<u>ACATCCAC</u>ACCCGTGATACGGGGTAATTATAGAGCA
CG

GCTGCACGCCCGAGTCCGAGTGCATAACAAAAATCCGCTTGGAGCCCGAGCCCGAGTCAATGATTATGGGTATTTA

TAGTCACGGCGCGTTGTTTATGAG<u>GGGGAAGT</u>GTGCGCTCTGCATATGTCTGATATCCA<u>CCGGATGTG</u>GATGTGGG
CG CG CG CG

GATGGTGATGCCGATGTACACACAATGCAGCCCTAGG<u>AGCATCCTT</u>GTGGCAGCTCTATAGC 746
CG

<u>Underlined</u> sites correspond to sites predicted by Philippakis et al. (2006).
